# Metabolic Signature of Dietary Iron Overload in a Mouse Model

**DOI:** 10.3390/cells7120264

**Published:** 2018-12-11

**Authors:** Chiara Volani, Giuseppe Paglia, Sigurdur V. Smarason, Peter P. Pramstaller, Egon Demetz, Christa Pfeifhofer-Obermair, Guenter Weiss

**Affiliations:** 1 Department of Internal Medicine II, Medical University of Innsbruck, Anichstrasse 35, 6020 Innsbruck, Austria; chiara.volani@i-med.ac.at (C.V.); Egon.demetz@i-med.ac.at (E.D.); Christa.pfeifhofer@i-med.ac.at (C.P.-O.); 2 Christian Doppler Laboratory for Iron Metabolism and Anemia Research, Medical University of Innsbruck, Anichstrasse 35, 6020 Innsbruck, Austria; 3 Institute for Biomedicine, Eurac Research, Via Galvani 31, 39100 Bolzano, Italy; beppepaglia@gmail.com (G.P.); sigurdur.smarason@eurac.edu (S.V.S.); Peter.pramstaller@eurac.edu (P.P.P.)

**Keywords:** iron, metabolomics, VAMS, mitochondria, glucose, urea cycle, oxidative stress

## Abstract

Iron is an essential co-factor for several metabolic processes, including the Krebs cycle and mitochondrial oxidative phosphorylation. Therefore, maintaining an appropriate iron balance is essential to ensure sufficient energy production and to avoid excessive reactive oxygen species formation. Iron overload impairs mitochondrial fitness; however, little is known about the associated metabolic changes. Here we aimed to characterize the metabolic signature triggered by dietary iron overload over time in a mouse model, where mice received either a standard or a high-iron diet. Metabolic profiling was assessed in blood, plasma and liver tissue. Peripheral blood was collected by means of volumetric absorptive microsampling (VAMS). Extracted blood and tissue metabolites were analyzed by liquid chromatography combined to high resolution mass spectrometry. Upon dietary iron loading we found increased glucose, aspartic acid and 2-/3-hydroxybutyric acid levels but low lactate and malate levels in peripheral blood and plasma, pointing to a re-programming of glucose homeostasis and the Krebs cycle. Further, iron loading resulted in the stimulation of the urea cycle in the liver. In addition, oxidative stress was enhanced in circulation and coincided with increased liver glutathione and systemic cysteine synthesis. Overall, iron supplementation affected several central metabolic circuits over time. Hence, in vivo investigation of metabolic signatures represents a novel and useful tool for getting deeper insights into iron-dependent regulatory circuits and for monitoring of patients with primary and secondary iron overload, and those ones receiving iron supplementation therapy.

## 1. Introduction

Iron is an essential element for human life [1,2,3] and also a fundamental catalytical co-factor for several enzymes, including aconitase in the Krebs cycle and electron transfer system (ETS) complexes in mitochondria [4,5].

Hence, an impairment of iron homeostasis, in the form of iron deficiency or iron overload, not only leads to increased production of reactive oxygen species (ROS) [1,6,7] but also to inappropriate energy availability. For instance, in iron overload disorders, excess iron accumulates in vital organs, like the liver [8], where it affects biochemical processes and mitochondrial respiratory capacity, as we have recently shown [9]. Altogether, these perturbations result in inadequate energy supply and may explain the phenotype of fatigue often reported by hemochromatosis patients [10].

Therefore, maintaining the iron balance is fundamental to preserve multiple enzymatic pathways ensuring energy requirements [11], and represents a public health concern [12].

Nevertheless, the impact of iron homeostasis perturbations on the overall body metabolic profiles (the metabolome) have not been explored thus far.

Metabolomics aims to comprehensively study metabolism, through the identification and quantification of small molecules, defined metabolites that reflect the phenotype investigated. As such, metabolomics is a dynamic approach of multiple biochemical pathway analysis, that also takes into account genetics, aging, environmental factors and lifestyle, thus allowing to identify specific metabolic signatures typical of a phenotype [13,14,15]. Metabolomic analysis can be performed in different tissues [16] and biofluids [17,18], and in combination with the information coming from the periphery tissue metabolites, it offers the possibility to better integrate and characterize systemic and tissue specific regulation of enzymatic pathways.

However, investigating peripheral metabolites in a mouse model is still challenging, especially when blood should be collected overtime, given the limited amount of blood that can be taken. Back in the 1960s Guthrie and Susi developed a system for the screening of phenylketonuria in newborn infants where only a few drops of whole blood, collected on filter paper, called dried blood spots (DBS), were sufficient for the diagnosis [19,20]. Nowadays, the use of DBS is very common in clinics [21,22,23] nevertheless its application is limited by several factors, including the influence of hematocrit (HCT), the volume of the blood spotted onto the filter paper, and spot homogeneity [17].

We have recently validated the use of a commercially available tool, allowing for volumetric absorptive microsampling (VAMS), in combination with a mass spectrometry (MS)-based untargeted metabolomics workflow [17]. This technology, similar to DBS sampling, is a novel system that allows accurate and representative single-drop blood (10 µL) collection [24,25] and overcomes the issues with hematocrit. 

Moreover, considering the minimal amount of blood required, using such approach in animal studies supports the principle of the three Rs (3Rs) by reducing animal sample size and minimizing animal pain [26].

To date, there are no studies combining VAMS and untargeted metabolomics in animal models. Therefore, in the current study we first aimed to test the suitability of this technology in a mouse model and then to investigate the metabolic signature driven by dietary iron overload. Giving the clinical importance of iron overload disorders, metabolic signature of altered iron homeostasis was investigated to identify circulating biomarkers that correlate with tissue dysfunction.

## 2. Materials and Methods

### 2.1. Chemicals

All materials were obtained from Sigma-Aldrich (Seelze, Germany), unless stated otherwise. Acetonitrile was purchased from VWR International (Radnor, PA, USA). Water was obtained from a Milli-Q water purification system equipped with a LC-Pak polisher (Merck, Darmstadt, Germany). All chemicals and solvents were of analytical grade or higher purity. The metabolomic amino acid mix standard, containing isotopically labeled amino acids, was purchased from Cambridge Isotope Laboratories (Tewksbury, MA, USA). 

### 2.2. Animal Models

Wild-type C57BL/6 animals were maintained and bred in the central animal facility at the Medical University of Innsbruck (Austria) under specific pathogen-free conditions. All animal experiments were performed in accordance with national and European guidelines and were reviewed and authorized by the committee on animal experiments (Federal Ministry of Science, Research and Economy-BMBWF-66.011/0035-WF/V/3b/2018). At the age of 10-weeks, male mice were randomly assigned either to the control group or the treatment group. The control group received a standard chow diet (n = 3; 180 mg of iron/kg) (Sniff, Soest, Germany), the treatment group received a diet supplemented with 25 g/kg of carbonyl iron (n = 5) (Altromin, Lage, Germany), for 14 days, respectively. At days 0, 7 and 14, blood was collected by means of VAMS (further description is included in the appropriate section). At day 14, animals were sacrificed, plasma heparin and liver tissue were collected for metabolomic analyses (description in the appropriate section).

### 2.3. Blood VAMS 

MITRA VAMS devices were purchased from Neoteryx (Torrance, CA, USA). One drop of mouse blood was absorbed onto VAMS devices at three different time points and processed as described in the appropriate section.

### 2.4. Blood, Plasma and Liver Metabolite Extraction

Whole blood was collected by means of VAMS and metabolites were extracted as previously described [17]. Briefly, VAMS tips were dried for 2 h and stored at −80 °C until metabolomics analysis. Next, VAMS were inserted into Eppendorf tubes and extracted with 200 µL of extraction solution containing the following internal standards: Alanine ^13^C_3_^15^N (0.9 μg/mL), arginine ^13^C_6_^15^N_4_ (1.8 μg/mL), aspartic acid ^13^C_4_^15^N (1.3 μg/mL), cystine ^13^C_6_^15^N_2_ (1.2 μg/mL), glutamic acid ^13^C_5_^15^N (1.5 μg/mL), glycine ^13^C_2_^15^N (0.8 μg/mL), histidine ^13^C_6_^15^N_3_ (1.6 μg/mL), isoleucine ^13^C_6_^15^N (1.3 μg/mL), leucine ^13^C_6_^15^N (1.3 μg/mL), lysine ^13^C_6_^15^N_2_ (1.5 μg/mL), methionine ^13^C_5_^15^N (1.5 μg/mL), phenylalanine ^13^C_9_^15^N (1.7 μg/mL), proline ^13^C_5_^15^N (1.2 μg/mL), serine ^13^C_3_^15^N (1.1 μg/mL), threonine ^13^C_4_^15^N (1.2 μg/ mL), tyrosine ^13^C_9_^15^N (1.8 μg/mL), and valine ^13^C_5_^15^N (1.2 μg/mL).

Samples were sonicated for 15 min and then vortexed at 1200 rpm for 60 min, both at 20 °C. VAMS tips were removed, and centrifuged at 1800× *g* for 10 min at 20 °C. To remove proteins, a protein removal plate (Sirocco Waters Corporation, Milford, MA, USA) was used, where the supernatant was filtered by using a positive pressure-96 processor applying 12 psi pressure (Waters Corporation, Milford, MA, USA). The extract was evaporated to dryness under vacuum at 35 °C for 120 min in a vacuum evaporator (EZ-2 vacuum evaporator, Genevac, Ipswich, UK). 

To obtain plasma, whole blood was collected in heparin tubes, and subsequently centrifuged at 8000 rpm for 8 min at 4 °C. Plasma samples were stored at −80 °C until the metabolomic analysis. Metabolites were obtained adding 200 μL of extraction solution to 50 μL of plasma. The extraction solution consisted of acetonitrile (100%) and internal standards in the concentrations described above. Samples were then filtered through a protein removal plate (Sirocco, Waters Corporation, Milford, MA, USA) and the extract was evaporated to dryness under vacuum at 35 °C for 120 min in a vacuum evaporator (EZ-2 vacuum evaporator, Genevac, Ipswich, UK). 

Around 30–50 mg of frozen liver samples were homogenized in ice-cold methanol (100%). 50 µL of this homogenate was added to 200 μL of extraction solution, proteins were removed as described above and the extracted metabolites were evaporated to dryness. 

Finally, all samples were reconstituted with 150 μL of acetonitrile/water (50:50, *v*/*v*) solution.

Quality control (QC) samples were obtained were obtained by pooling together small aliquots (15 µL) from each sample.

### 2.5. Ultra-High Performance Liquid Chromatography (UHPLC) Combined with MASS Spectrometry (MS)

All samples were analyzed using the metabolomics workflow previously described [17]. Briefly, ultra high-performance liquid chromatography (UHPLC) (Agilent 1290; Agilent Technologies, Santa Clara, CA, USA) was coupled to a Q-TOF mass spectrometer (TripleTOF 5600+; AB Sciex, Foster City, CA, USA). The chromatographic separation was achieved by hydrophilic interaction liquid chromatography (HILIC) using an Acquity BEH amide, 100 × 2.1 mm column (Waters Corporation, Milford, MA, USA). 

Acetonitrile + 0.1% formic acid was used as mobile phase A and water + 0.1% formic acid as mobile phase B. The injection volume was set at 5 μL, and the flow rate at 0.6 mL/min. The following linear gradients were used: 0 min 95% A and 1 min 95% A, 4 min 30% A and 5 min 30% A, and 5.1 min 95% A and 8 min 95% A. 

The mass spectrometer operated in full scan mode in the mass range from 50 to 1000 *m*/*z* and an accumulation time of 250 ms. In ESI+ mode, the source temperature was set at 700 °C, the declustering potential at 30 V, the collision energy at 6 V, the ion spray voltage at 5120 V, the curtain gas at 25 psi, and the ion source gases 1 and 2 at 60 psi. In ESI-mode, the source temperature was set at 650 °C, the declustering potential at −45 V, the collision energy at −6 V, the ion spray voltage at −3800 V, the curtain gas at 25 psi, and the ion source gases 1 and 2 at 30 psi. The instrument was mass calibrated by automatic calibration infusing the Sciex Positive Calibration Solution (part no. 4460131, AB Sciex, Foster City, CA, USA) for positive mode and Sciex Negative Calibration Solution (part no. 4460134, AB Sciex, Forster City, CA, USA) for negative mode, after every two sample injections. Samples were then analyzed in a randomized order, and pooled QC samples were injected every eight samples.

### 2.6. Data Analysis

Data were first converted to mzML, using ProteoWizard MS Convert [27] and then processed in R (v 3.3.3) using the XCMS package [28]. Pooled QCs and total ion current (TIC) were used to check the intra- and inter-batch variability, as applicable. We applied an acceptance of 30%. Therefore, features/metabolites with RSD higher than 30% in pooled QCs were removed and not considered further in data analysis. Failed injections were checked by monitoring the intensity of the spiked labeled amino acid standards.

Metabolites were identified by verifying retention time, accurate mass, and tandem mass spectrometry against our in-house and/or online databases, including the Human Metabolome Database (HMDB) [29] and the metabolomics database (METLIN) [30]. Among the annotated metabolites, we identified 2-hyroxybutyric acid, however we cannot exclude the presence of the isomer 3-hyroxybutyric acid, which might not be separated by our chromatographic system. From now on, we will refer to this metabolite as 2-/3-hyroxybutyric acid. Tentatively identified metabolites were integrated using MultiQuant 3.0 (Sciex, Foster City, CA, USA). Extracted ion chromatograms (EICs) were extracted using a 0.02 mDa window centered on the expected *m*/*z* for each targeted compound. 

Data were normalized by the sum of the features and then log transformed and scaled by using Pareto scaling before principal component analysis (PCA) using MetaboAnalyst (version 4.0) [31].

## 3. Results

We recently developed a method based on VAMS for collection, storage and extraction of peripheral blood metabolites, suitable for a mass spectrometry (MS)-based metabolomics workflow [17].

Despite the minimal amount of blood required, this technology offers the advantage of multiple sampling that is of great convenience in case of animal research. Hence, in this work we applied this workflow to study metabolic changes over time in mice exposed to dietary iron loading.

Accordingly, we compared 10-week-old male C57BL/6 animals receiving a normal diet and an iron enriched diet. We collected blood before the start of the diet (at day 0), and subsequently at day 7 and 14, along with analysis of plasma heparin and liver tissue at day 14. Details of the experimental design are reported in Materials and Methods and in Figure S1.

### 3.1. Dietary Iron Overload Changes the Metabolic Signature over the Time

**Figure 1:**
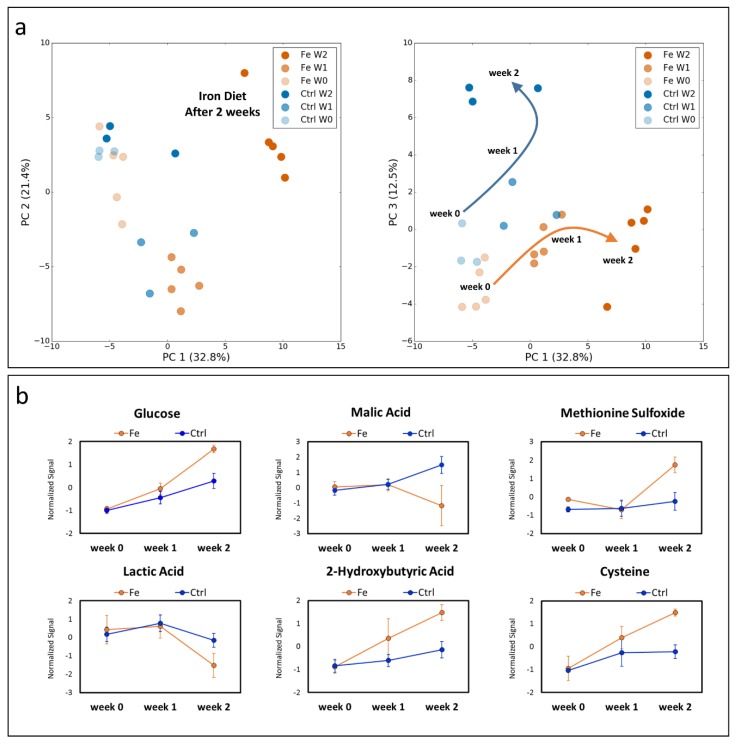
Metabolic profiles of iron diet fed mice over a period of two weeks. Principal component analysis (PCA) (**a**) of peripheral blood metabolites shows metabolic profile changes overtime and distinct metabolic signatures between dietary iron challenged and control animals. Specific profiles of selected metabolites (**b**) shows their quantitative changes over time. Controls (Ctrl, blue, n = 3), high-iron diet (Fe, orange, n = 5). Statistics: 2-way ANOVA was performed, a *p*-value < 0.05 was considered to be significant.

To evaluate the metabolic changes over time associated with dietary iron loading we first analyzed whole blood which was collected by means of VAMS before starting the diet (day 0), after one (day 7) and two weeks (day 14) of the diet. Data were initially visualized by applying a principal component analysis (PCA) (Figure 1a). The first principal component (PC1) and the second component (PC2) explained 32.8% and 21.4% respectively of the total variance of the dataset, and clearly show that mice receiving an iron enriched diet for two weeks cluster from control animals. When plotting PC1 versus PC3 (12.5% of the total variance) we noticed that iron challenge affected the metabolic profile of mice in a time specific manner generating, a specific metabolic signature which was different from that obtained in controls (Figure 1a).

**Figure 2:**
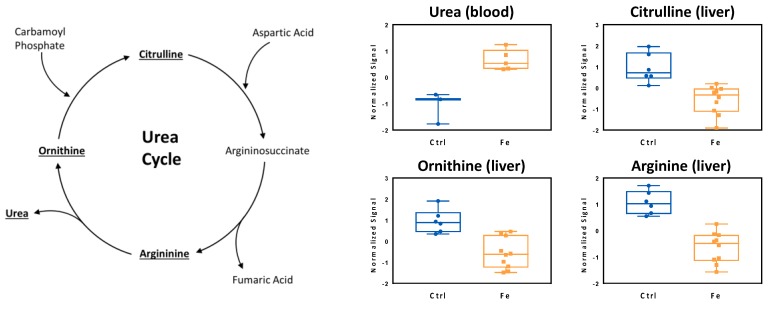
High iron diet induces the urea cycle in the liver, thereby enhancing the levels of urea in blood. Higher levels of the metabolite urea were detected in the blood and this was concomitant with an enhanced urea cycle, driving to lower levels of ornithine, citrulline, and arginine in the liver. Controls (Ctrl, blue, n = 3), high-iron diet (Fe, orange, n = 5). In the liver, each sample was run in duplicates. Statistics: Student *t*-test was performed, a *p*-value < 0.05 was considered to be significant.

The specific trend observed in the PCA was due to significant changes of the metabolome over time. For instance, Figure 1b shows the changes in glucose, malic acid, methionine sulfoxide, lactic acid, 2-/3-hydroxybutyric acid and cysteine levels over time, indicating that these profiles are not comparable with the ones obtained from controls (Figure 1b), suggesting that iron overload affects systemic metabolism. The complete list of metabolites that changed upon iron supplementation is described in Table 1. In addition, animals fed a high iron diet displayed increased urea blood levels indicating a dysregulated urea cycle in the liver (Figure 2). Accordingly, the liver metabolome was characterized by decreased levels of ornithine, citrulline and arginine.

### 3.2. Validation of the Systemic Metabolic Signature

**Figure 3:**
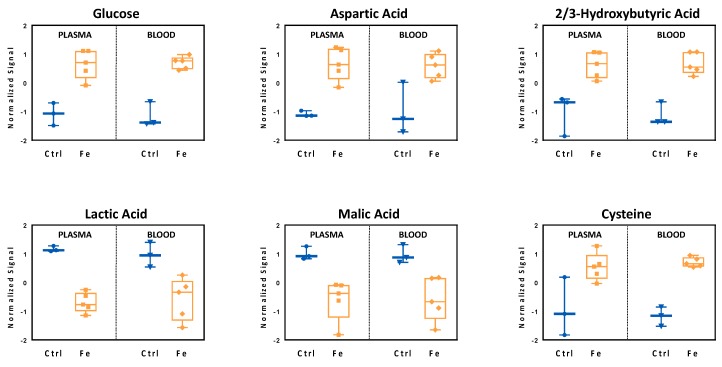
Plasma and blood metabolite profiles were compared and highlight alterations of glucose homeostasis as well as oxidative stress induction by iron challenge. Comparison of normalized signals of selected metabolites extracted from plasma and blood show that the profiles are very similar. Controls (Ctrl, blue, n = 3), high-iron diet (Fe, orange, n = 5). Statistics: Student *t*-test was performed, a *p*-value < 0.05 was considered to be significant.

To validate the metabolic signatures seen in peripheral blood, we repeated the metabolomics analysis on plasma samples obtained from the same animals after two weeks of diet (Figure 3, Table 2).

Plasma is routinely preferred for clinical chemistry laboratory tests, considering the absence of blood cells, making such a matrix less complex than whole blood. Even if plasma metabolome does not contain metabolites coming from blood cells [32,33,34], the overall metabolic signature should be similar to the one detected in whole blood. Indeed, we found a similar metabolic signature between blood and plasma confirming the findings of the VAMS analysis (Figure 3). In particular, focusing on six metabolites, glucose, lactate, malic acid, aspartic acid, 2-/3-hydroxybutyric acid and cysteine (Figure 3), we observed higher levels of cysteine and 2-/3-hydroxybutyric (2/3HBA) acid in animals receiving the high-iron diet, indicating a possible compensatory mechanism to increased oxidative stress. 2-hydroxybutyric acid, a metabolite primarily originating from mammalian hepatic tissues is produced under stress conditions (lipid oxidation, oxidative stress) and in case of impaired glucose regulation [35,36,37]. Interestingly, iron overload, blood and plasma glucose levels increased and were characterized by a concomitant reduction in lactic acid, suggesting an impairment in glucose homeostasis or alternatively an iron-dependent stimulation of the Krebs cycle, leading to the use of other substrates rather than glucose [4,38].

### 3.3. Analysis of Liver Metabolome Reflects Iron-Induced Changes Observed in the Circulation

The significant changes of malic and aspartic acid levels observed in blood and plasma (Figure 3) might reflect alterations in mitochondrial function or energy utilization in tissues [39], as these two metabolites are involved in the malate-aspartate shuttle, a biochemical system which ensures mitochondrial fitness. Accordingly, we have recently reported that iron overload affects mitochondrial respiratory capacity in the liver, the organ deputed for the storage of excess iron and orchestration of systemic iron homeostasis via hepcidin [9] (Figure S2). For this reason, we next investigated if the liver metabolome could explain the systemic metabolic signature. 

**Figure 4:**
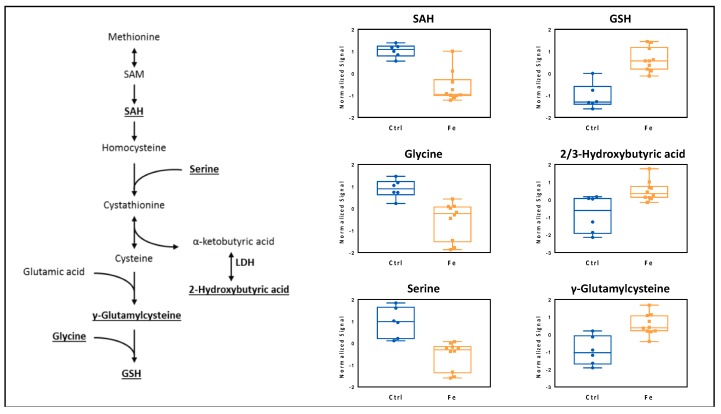
Metabolic profiling of the liver upon iron challenge. Dietary iron loading affects the transmethylation pathway, driving glutathione synthesis and production of 2-/3-hydroxybutyric acid. SAM: S-adenosyl-L-methionine; SAH: S-adenosylhomocysteine; LDH: Lactate dehydrogenase; GSH: Glutathione. Controls (Ctrl, blue, n = 3), high-iron diet (Fe, orange, n = 5). Each sample was run in duplicates. Statistics: Student *t*-test was performed, a *p*-value < 0.05 was considered to be significant.

On one hand, iron supplementation triggered liver metabolic remodeling to cope with oxidative stress that primarily enhanced transmethylation pathways, resulting in increased glutathione synthesis (Figure 4, Table 3). On the other hand, dietary iron loading resulted in downregulation of carnitines (Table 3). The latter is likely due to carnitines consumption as indicated by the low levels of the carnitines detected both in the periphery and in the liver. In this setting, carnitines were probably used not only as antioxidant molecules, but also as response molecules to the systemic glucose imbalance [40], given the high levels of glucose detected in plasma and blood (Figure 3).

Moreover, in accordance with the results obtained in plasma and blood, increased 2-/3-hydroxybutyric acid levels were also found in the liver.

## 4. Discussion

This is the first study that combines the use of VAMS technology to a metabolomics workflow for the investigation of the effects of dietary iron overload on metabolic profiles. Moreover, this study provides new information on the significance of metabolite levels found in plasma and whole blood related to metabolic changes observed in organs, specifically in the liver.

The latter is the central organ for several physiological and metabolic processes, including iron homeostasis. To that, despite the knowledge gained so far, the overall mechanisms leading to patient’s subclinical problems related to insufficient energy metabolism are still insufficiently understood [1,4,7,11]. For this reason, exploring the effects of iron homeostasis imbalances and the underlying metabolic rearrangements remain a public health concern. In particular, a better understanding of such processes would be of great importance for patient handling, refinement of genetic and transfusional iron loading, and to monitor intravenous iron therapy in order to prevent tissue damage or systemic problems caused by spatial-temporal accumulation of iron [1,7,41,42]. In this context, the possibility to use VAMS devices would offer the advantage to easily screen subjects at risk, and subsequently follow the affected ones.

In accordance with previous reports from human studies [43,44], in this study we found that iron overload caused a metabolic remodeling that primarily involves a compensatory response to increased oxidative stress. Here we also found, that in response to iron overload, concentrations of blood antioxidant metabolites, such as methionine sulfoxide, glutathione, cysteine, were increased. Under such metabolic stress conditions, 2-/3-hydroxybutyric acid, which is a byproduct of cystathionine cleavage to cysteine in the methionine-to-glutathione pathway, can be released [45]. Indeed, 2-/3-hydroxybutyric acid was found to be associate with excess glutathione demand and disrupted mitochondrial energy mechanisms, occurring during persistent oxidative stress [35].

In the current study, dietary iron overload increased liver 2-/3-hydroxybutyric acid synthesis and its excretion into blood and plasma, indicating an augmented hepatic glutathione synthesis, necessary to cope with the ongoing oxidative stress. 

Moreover, besides bearing detoxification functions, this metabolite has been also reported as an early biomarker for insulin resistance and glucose intolerance in a non-diabetic population [35,36,45].

Interestingly, animals receiving the high iron supplement showed higher glucose levels. This is in agreement with the literature that reports an association between hyperferritenemia and glucose [44,46,47,48]. Nevertheless, the exact mechanisms that link iron to diabetes development still remain to be elucidated.

With regard to that, our experiments suggest two possible explanations. Firstly, iron loading might act as an activator of the Krebs cycle which is fueled by other substrates rather than glucose; in this way the number of molecules of glucose needed for energy production are reduced and also the ones, eventually, used for lactate production [4].

Secondly, iron loading affects carnitine levels. Carnitines are small molecules that carry out a plethora of functions. Besides being involved in the transport of long-chain fatty acids from the cytosol into the mitochondria, carnitines play an important role in energy production, through the coordination of the activity of several enzymes of the Krebs cycle, beta oxidation, urea cycle and gluconeogenesis [40]. If their balance is maintained correctly, carnitines improve glucose tolerance, on the contrary low levels of carnitines are associated with diabetic complications [40,49,50]. 

Accordingly, high iron overload led to carnitines consumption as shown by the low levels of carnitines detected both in the periphery and in the liver. In this setting, carnitines may be employed both as antioxidant molecules and seen as a metabolic response to systemic glucose imbalance [40].

Altogether, these results support the association between iron loading and altered glucose homeostasis already described in humans [36]. Moreover, in accordance to what we have previously shown [9], dietary iron overload caused not only mitochondrial impairment, but also changes to metabolic remodeling, that primarily implies compensatory mechanisms to oxidative stress.

Some of the presented metabolites changing in blood during iron overload, such as glucose, 2-/3-hydroxybutyric, malic acid, aspartic acid, lactic acid and cysteine might represent potential biomarkers for iron imbalances and tissue iron loading. Nevertheless, to confirm these findings further in vivo studies with larger sample sizes are necessary.

Finally, although plasma is usually preferred for diagnostic purposes, this work demonstrates the advantage of using VAMS technology, considering the minimal amount of blood required and the avoidance of additional pre-analytical procedures to obtain plasma. Moreover, considering that for animal research, plasma is often the limiting step for exploratory experiments, we strongly recommend the use of such a device. Our data not only uncovers regulatory metabolic circuits affected by dietary iron loading but also suggests that determination of metabolites in the periphery provide a suitable reflection of comparable metabolic changes in the liver. 

## 5. Conclusions

In conclusion, metabolomics offers an advantageous platform to monitor metabolic changes occurring during iron overload. In particular, the use of VAMS technology represents a reliable and useful tool for blood sampling in animal research, in accordance with the principles of reduction and refinement (two of the 3Rs principles). Specifically, VAMS technology allows multiple sampling, reducing the number of animals needed for the study on one side, as well as easy blood collection, diminishing the pain and distress of the animals.

Moreover, the combination of VAMS with MS-based untargeted metabolomics facilitates the collection of time-course metabolomics data, allowing to study changes in the metabolic signature over the time during a perturbation.

In the current study, we found a specific blood signature after iron supplementation that involves changes in the systemic redox state. In addition, iron overload affects glucose homeostasis, suggesting ongoing metabolic remodeling, which may be linked to the mitochondrial dysfunction we previously reported [9], and supporting the association between iron loading and dysregulated glucose homeostasis reported in humans [36].

For this reason, further investigations of the metabolic signature of subjects suffering from primary or secondary iron overload will provide new insights into the association between iron imbalance and glucose homeostasis. 

## 6. Limitations of the Study

The animal model presented in this study was used to explore the metabolic signature of after high iron supplementation. Nevertheless, this model presents some limitations, including the supra-physiological concentrations of iron in the diet. Moreover, the liver was not perfused, therefore some of the metabolite concentrations detected in the liver might have actually been influenced by residual blood in the liver.

## Figures and Tables

**Table 1:** Metabolites significantly changing (increasing ↑, decreasing ↓) in peripheral blood after two weeks of a high-iron diet as compared to animals on a control diet. Controls (Ctrl, blue, n = 3), high-iron diet (Fe, orange, n = 5). Statistics: Student *t*-test was performed, fold-change was calculated and a *p*-value < 0.05 was considered to be significant.

Metabolite	HMDB	Fold Change	*p* Value	Trend in Fe
AMP	HMDB00045	3.9	7.9 × 10^−6^	↑
ADP	HMDB01341	2.9	8.1 × 10^−4^	↑
2/3-Hydroxybutyric acid	HMDB0000008/HMDB00357	2.5	7.0 × 10^−4^	↑
Glucose	HMDB00122	2.5	1.8 × 10^−4^	↑
N1-Methyl-2-pyridone-5-carboxamide	HMDB0004193	2.4	1.3 × 10^−3^	↑
Gluconic acid	HMDB00625	2.3	3.0 × 10^−3^	↑
l-Aspartyl-4-phosphate	HMDB0012250	2.3	1.2 × 10^−2^	↑
Hydroxyproline	HMDB00725	2.3	6.7 × 10^−3^	↑
Methionine sulfoxide	HMDB0002005	2.1	8.6 × 10^−4^	↑
l-Aspartic acid	HMDB00191	2.1	1.4 × 10^−2^	↑
γ-Glutamylcysteine	HMDB0001049	2.0	3.4 × 10^−3^	↑
l-Cysteine	HMDB00574	1.9	3.6 × 10^−5^	↑
Hydroxyisocaproic acid	HMDB0000746	1,8	8.0 × 10^−3^	↑
Pyroglutamic acid	HMDB0000267	1.7	2.6 × 10^−3^	↑
ADP-Glucose	HMDB06557	1.7	1.7 × 10^−2^	↑
Adenosine	HMDB00050	1.7	2.4 × 10^−2^	↑
2-Phosphoglyceric acid	HMDB00362	1.6	1.9 × 10^−5^	↑
Phosphorylethanolamine	HMDB00224	1.5	3.8 × 10^−4^	↑
GMP	HMDB01397	1.5	2.5 × 10^−2^	↑
UDP-glucose	HMDB00286	1.5	5.3 × 10^−5^	↑
UDP-N-acetylglucosamine	HMDB0000290	1.5	1.1 × 10^−4^	↑
Glucose 6-phosphate	HMDB01401	1.5	2.3 × 10^−4^	↑
Acetylmethionine	HMDB11745	1.5	2.3 × 10^−3^	↑
l-Threonine	HMDB00167	1.5	1.7 × 10^−2^	↑
CDP-ethanolamine	HMDB01564	1.5	3.7 × 10^−3^	↑
N-Acetyl-beta-alanine	HMDB0061880	1.4	2.4 × 10^−3^	↑
Glyceric acid	HMDB00139	1.4	4.6 × 10^−2^	↑
Phosphoenolpyruvic acid	HMDB00263	1.4	2.9 × 10^−3^	↑
2-Octenedioic acid	HMDB0000341	1.4	5.0 × 10^−2^	↑
Pyruvic acid	HMDB00243	1.4	1.2 × 10^−2^	↑
l-Lysine	HMDB00182	1.4	8.5 × 10^−5^	↑
Pipecolic acid	HMDB00070	1.4	8.1 × 10^−5^	↑
PABA	HMDB01392	1.4	2.8 × 10^−2^	↑
l-Proline	HMDB00162	1.3	4.3 × 10^−2^	↑
Glycolic acid	HMDB00115	1.3	3.2 × 10^−2^	↑
Glutaric acid	HMDB00661	1.3	3.5 × 10^−3^	↑
l-Glutamic acid	HMDB00148	1.3	1.2 × 10^−2^	↑
l-Asparagine	HMDB00168	1.2	2.9 × 10^−2^	↑
Creatinine	HMDB00562	1.2	1.7 × 10^−2^	↑
Urea	HMDB0000294	1.2	2.3 × 10^−3^	↑
ADMA/SDMA	HMDB0001539/HMDB0003334	1.2	3.3 × 10^−2^	↑
Choline	HMDB00097	1.1	1.3 × 10^−3^	↑
N6,N6,N6-Trimethyl-L-lysine	HMDB0001325	1.1	2.7 × 10^−2^	↑
IMP	HMDB00175	0.3	1.1 × 10^−2^	↓
N4-Acetylcytidine	HMDB0005923	0.4	4.5 × 10^−2^	↓
l-Acetylcarnitine	HMDB00201	0.4	5.0 × 10^−6^	↓
Stearoylcarnitine	HMDB0000848	0.5	1.8 × 10^−2^	↓
α-ketoisovaleric acid	HMDB00019	0.5	9.9 × 10^−3^	↓
Propionylcarnitine	HMDB0000824	0.7	3.5 × 10^−2^	↓
Malic acid	HMDB00156	0.7	1.8 × 10^−2^	↓
Ornithine	HMDB00214	0.7	4.4 × 10^−2^	↓
(Iso)leucine	HMDB00172	0.7	2.6 × 10^−2^	↓
Decanoylcarnitine	HMDB0000651	0.7	2.4 × 10^−2^	↓
l-Arginine	HMDB00517	0.8	4.3 × 10^−3^	↓
l-Tryptophan	HMDB00929	0,8	9.5 × 10^−3^	↓
Lactic acid	HMDB00190	0.8	1.8 × 10^−2^	↓
l-Palmitoylcarnitine	HMDB0000222	0.8	2.2 × 10^−2^	↓
Pyrrolidonecarboxylic acid	HMDB0000805	0.9	4.4 × 10^−2^	↓

**Table 2:** Metabolites significantly changing (increasing ↑, decreasing ↓) in plasma after two weeks of a high-iron diet as compared to animals kept on a control diet. Controls (Ctrl, blue, n = 3), high-iron diet (Fe, orange, n = 5). Statistics: Student *t*-test was performed, fold-change was calculated and a *p*-value < 0.05 was considered to be significant.

Metabolite	HMDB	Fold Change	*p* Value	Trend in Fe
l-Aspartic acid	HMDB00191	3.4	2.2 × 10^−3^	↑
l-Cysteine	HMDB00574	2.2	3.0 × 10^−2^	↑
N1-Methyl-2-pyridone-5-carboxamide	HMDB0004193	2.1	1.2 × 10^−2^	↑
Hydroxyproline	HMDB00725	2.0	6.1 × 10^−3^	↑
2-Octenedioic acid	HMDB0000341	2.0	3.3 × 10^−2^	↑
Glucose	HMDB00122	1.9	2.3 × 10^−3^	↑
Hydroxyisocaproic acid	HMDB0000746	1.8	1.3 × 10^−3^	↑
Glutathione	HMDB0000125	1.7	2.7 × 10^−2^	↑
2/3-Hydroxybutyric acid	HMDB0000008/HMDB00357	1.7	6.5 × 10^−3^	↑
Pipecolic acid	HMDB00070	1.4	1.3 × 10^−2^	↑
l-Lysine	HMDB00182	1.4	8.2 × 10^−3^	↑
γ-Glutamylcysteine	HMDB0001049	1.4	4.0 × 10^−4^	↑
Choline	HMDB00097	1.4	1.0 × 10^−2^	↑
1-Methylhistidine	HMDB00001	1.3	3.2 × 10^−2^	↑
Adenosine	HMDB00050	1.3	8.1 × 10^−3^	↑
Acetylcarnosine	HMDB0012881	1.3	4.8 × 10^−2^	↑
l-Serine	HMDB00187	1.2	4.0 × 10^−2^	↑
l-Histidine	HMDB00177	1.2	1.1 × 10^−2^	↑
l-Acetylcarnitine	HMDB00201	0.3	8.0 × 10^−5^	↓
Propionylcarnitine	HMDB0000824	0.3	1.8 × 10^−3^	↓
Malic acid	HMDB00156	0.3	1.1 × 10^−2^	↓
IMP	HMDB00175	0.4	5.7 × 10^−3^	↓
Gamma-linolenyl carnitine	HMDB0006318	0.5	1.6 × 10^−2^	↓
Lactic acid	HMDB00190	0.7	1.2 × 10^−4^	↓

**Table 3:** Metabolites significantly changing (increasing ↑, decreasing ↓) in liver after two weeks of a high-iron diet as compared to control animals. Controls (Ctrl, blue, n = 3), high-iron diet (Fe, orange, n = 5). Each sample was run in duplicate. Statistics: student *t*-test was performed, fold-change was calculated and a *p*-value < 0.05 was considered to be significant.

Metabolite	HMDB	Fold Change	*p* Value	Trend in Fe
Ascorbic Acid	HMDB00044	2.0	0.0208	↑
ADP-Glucose	HMDB06557	1.7	0.0169	↑
Aconitic acid	HMDB0000072	1.7	0.0206	↑
γ-Glutamylcysteine	HMDB0001049	1.6	0.0010	↑
Ureidopropionic acid	HMDB00026	1.6	0.0159	↑
2/3-Hydroxybutyric acid	HMDB0000008/HMDB00357	1.6	0.0050	↑
Folic acid	HMDB00121	1.3	0.0402	↑
Glucosamine	HMDB01514	1.3	0.0164	↑
Hydroxyproline	HMDB00725	1.2	0.0138	↑
Glutathione	HMDB0000125	1.2	0.0000	↑
Taurine	HMDB00251	1.1	0.0140	↑
CDP-ethanolamine	HMDB01564	0.5	0.0350	↓
Propionylcarnitine	HMDB0000824	0.5	0.0015	↓
l-Aspartyl-4-phosphate	HMDB0012250	0.5	0.0086	↓
l-Serine	HMDB00187	0.5	0.0005	↓
Acetylhistidine	HMDB32055	0.5	0.0030	↓
Xanthosine	HMDB00299	0.5	0.0030	↓
l-Arginine	HMDB00517	0.5	0.0000	↓
N6-Methyllysine	HMDB0002038	0.5	0.0000	↓
l-Valine	HMDB00883	0.6	0.0050	↓
Hypoxanthine	HMDB00157	0.6	0.0492	↓
l-Proline	HMDB00162	0.6	0.0252	↓
Carnosine	HMDB00033	0.6	0.0003	↓
Creatinine	HMDB00562	0.6	0.0400	↓
IMP	HMDB00175	0.6	0.0024	↓
Allantoin	HMDB00462	0.6	0.0221	↓
Glycerol-3-Phosphate	HMDB00126	0.6	0.0043	↓
UDP-glucose	HMDB00286	0.6	0.0309	↓
Guanine	HMDB00132	0.6	0.0425	↓
Citrulline	HMDB00904	0.6	0.0007	↓
l-Carnitine	HMDB00062	0.6	0.0005	↓
Choline	HMDB00097	0.6	0.0183	↓
SAH	HMDB00939	0.6	0.0001	↓
Inosine	HMDB00195	0.6	0.0434	↓
Uric acid	HMDB00289	0.7	0.0470	↓
Creatine	HMDB00064	0.7	0.0462	↓
l-Acetylcarnitine	HMDB00201	0.7	0.0292	↓
l-Asparagine	HMDB00168	0.7	0.0104	↓
Glycerylphosphorylethanolamine	HMDB0000114	0.7	0.0037	↓
(Iso)leucine	HMDB00172	0.7	0.0018	↓
Ornithine	HMDB00214	0.7	0.0007	↓
Pentose-Phosphate	HMDB0000098/HMDB0001489/HMDB0001548	0.7	0.0297	↓
Glycine	HMDB00123	0.7	0.0018	↓
l-Phenylalanine	HMDB00159	0.8	0.0017	↓
l-Threonine	HMDB00167	0.8	0.0023	↓
l-Glutamine	HMDB00641	0.9	0.0398	↓

## Supplementary Materials

The following are available online at http://www.mdpi.com/2073-4409/7/12/264/s1. Figure S1: Experimental design to monitor metabolic changes during dietary iron overload, Figure S2: Dietary iron overload increases both plasma iron levels as well as liver expression of ferritin.
