# Supplementary material for: Metabolic Signature of Dietary Iron Overload in a Mouse Model
Source: Cells. 2018 Dec 11;7(12):264. doi: 10.3390/cells7120264 (PMC6315421; doi:10.3390/cells7120264)
Supplement: Supplementary file 1 [file cells-07-00264-s001.zip › cells-397827-SI.pdf]

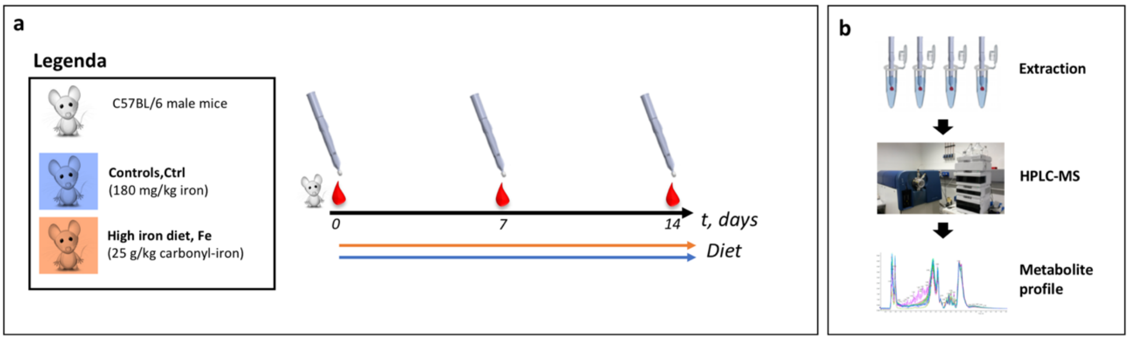

Figure S1. Experimental design to monitor metabolic changes during dietary iron overload. Ten-week-old C57BL/6 male animals (**a**) were randomized either to the control group, fed a normal diet (control animals), or to the treatment group, fed a high-iron diet (Fe animals). On day 0, day 7 and day 14 blood was sampled by means of volumetric absorptive microsampling (VAMS). At day 14 animals were sacrificed, liver and plasma were collected. Blood metabolites (**b**) were extracted and measured according to the method described [17]. Plasma and liver metabolites were also analyzed as described in the section Materials and Methods.

SUPPLEMENTARY Figure 2

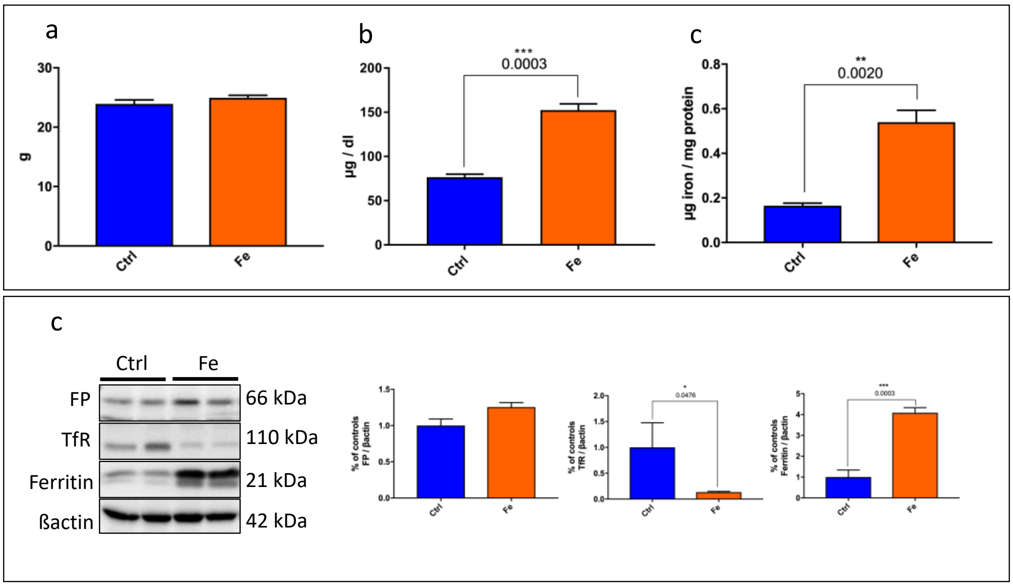

Figure S2. Dietary iron overload increases both plasma iron levels as well as liver expression of ferritin. Animal weight measured after two weeks of diet (a)**.** Plasma (b) iron and liver (c) iron content analysis. Liver (c) representative Western blot of the expression of iron-related proteins and quantitative analysis from all samples normalized to beta-actin and presented as % of the controls. FP: ferroportin; TfR: transferrin receptor. Controls (Ctrl, blue, n = 3), high-iron diet (Fe, orange, n = 5). Statistics: student t-test was performed, a p-value <0.05 was considered to be significant, the exact p-values are indicated in the graphs.
